# Metal Nanoparticles against Viruses: Possibilities to Fight SARS-CoV-2

**DOI:** 10.3390/nano11113118

**Published:** 2021-11-19

**Authors:** Marcelly Chue-Gonçalves, Giovana N. Pereira, Lígia C. Faccin-Galhardi, Renata K. T. Kobayashi, Gerson Nakazato

**Affiliations:** 1 Laboratory of Basic and Applied Bacteriology, Department of Microbiology, Biological Sciences Center, Londrina State University, Londrina 86057-970, Brazil; marcelly.chue@gmail.com (M.C.-G.); giovananicolete@gmail.com (G.N.P.); kobayashirkt@uel.br (R.K.T.K.); 2 Laboratory of Virology, Department of Microbiology, Biological Sciences Center, Londrina State University, Londrina 86057-970, Brazil; lgalhardi@uel.br

**Keywords:** nanoparticles, coronavirus, personal protective equipment, antiviral, COVID-19, SARS-CoV-2

## Abstract

In view of the current Coronavirus Disease 2019 (COVID-19) pandemic outbreak, the research community is focusing on development of diagnostics, treatment, and vaccines to halt or reverse this scenario. Although there are already various vaccines available, adaptive mutations in the SARS-CoV-2 genome can alter its pathogenic potential and, at the same time, increase the difficulty of developing drugs or immunization by vaccines. Nanotechnology carries a potential to act in all stages in fighting this viral disease, with several possibilities of strategies such as applying nanoparticles directly as antivirals in delivery systems against these viruses or incorporating them in materials, with power of achievement in therapeutics, vaccines and prevention. In this paper, we review and bring insights of recent studies using metal nanocomposites as antivirals against coronavirus and structurally similar viruses.

## 1. Introduction

Over number and variety, antiviral drugs are still a limited group. It is a huge challenge to develop an antiviral drug that does not harm the host due to dependence of the host cell machinery for viral replication [1]. Because of being obligate intracellular pathogens, reducing adverse effects and increasing selective toxicity are crucial points when developing a new antiviral. Other aspects that also turn arduous the development of potential new molecules as antivirals are limitations of in vitro assays, low availability of animal models capable of simulating human infections and the resistance that some strains can present. The versatility of nanocomposites makes them considered as a powerful tool in fighting infections and in prevention against viruses because they have unique physical and chemical properties that can be exploited and controlled in the process of synthesis. Metallic nanoparticles can generally be less than one micrometer in size, a determining factor for antimicrobial action to be attributed primarily to the area/volume ratio, and depending on the nanomaterial size, it can interfere with the viral replication inside the host cell as an advantage over other non-nanomaterials.

The malleable optical absorption spectra of metallic nanoparticles due to surface plasmon resonance is a huge advantage of using nanometals in bioconjugation, which is important when it comes to antiviral nanocarriers or drug stabilization [2]. Because its composition table is an important factor for the understanding of its antiviral action, this review will approach metal nanocomposites because they can activate production of hosts reactive oxygen species (ROS) through the ion release, a strategy well studied for antibacterial purposes [3,4].

Although the use of metallic nanoparticles can result in the emergence of some side effects, such as unwanted tissue interactions or increased inflammation, the multifunctionality of nanoparticles has gained prominence in strategies to face the crisis caused by COVID-19, as progress in the use of metallic NPs as antiviral agents advanced rapidly due to the ability of metals to “attack” multiple virus targets, with minimal impact on subsequent resistance development [5,6,7,8,9,10,11].

In this review, general aspects of the pandemic and the SARS-CoV-2 virus will be addressed, as well as epidemiological data in a global panorama. Furthermore, because there are few studies that use nanometals as antiviral agents against coronavirus, here we will discuss also nanometals against other structurally similar viruses. Along with SARS-CoV, MERS-CoV and coronaviruses that infect animals, other enveloped and RNA viruses will be discussed below as targets for metallic nanomaterials.

## 2. SARS-CoV-2

In January 2020, the Chinese Center for Disease Control and Prevention (CCDC) documented that 27 cases of pneumonia admitted in December 2019 were caused by a virus, later identified as Severe Acute Respiratory Syndrome 2 (SARS-CoV-2) virus, which was soon classified as the causative agent of COVID-19 by the World Health Organization (WHO) [12]. The new coronavirus, SARS-CoV-2, responsible for COVID-19, is a virus from the Betacoronavirus genus, belonging to the *Coronaviridae* family and *Coronavirinae* subfamily composed of three genera: *Alphacoronavirus*, *Betacoronavirus* and *Gammacoronavirus*. Among the 17 viruses belonging to *Coronavirinae*, seven are capable of infecting humans, being human coronavirus 229E (HCoV-229E) and human coronavirus NL63 (HCoV-NL63) belonging to the genus *Alphacoronavirus*, while human coronavirus OC43 (HCoV-OC43), human coronavirus HKU1 (HCoV-HKU1), Middle East Respiratory Virus (MERS-CoV), SARS-CoV and SARS-CoV-2 belong to the genus *Betacoronavirus* [13,14]. In the same subfamily, the genus *Alphacoronavirus* also presents epidemiological relevance because some of them can cause animal nonhuman infections such as in felines (Feline Coronavirus–FcoV) and in porcine (Transmissible Gastroenteritis Virus–TGEV; Porcine Epidemic Diarrhea Virus–PEDV) [14].

While HCoV-229E, HCoV-NL63, HCoV-OC43, and HCoV-HKU1 are related to mild colds attributed to infections of the upper respiratory system, SARS-CoV, MERS-CoV and SARS-CoV-2 cause severe respiratory disease with complex pathophysiology, which can be associated with multiple organ failure, sepsis and death [15,16,17,18,19,20].

**Figure 1:**
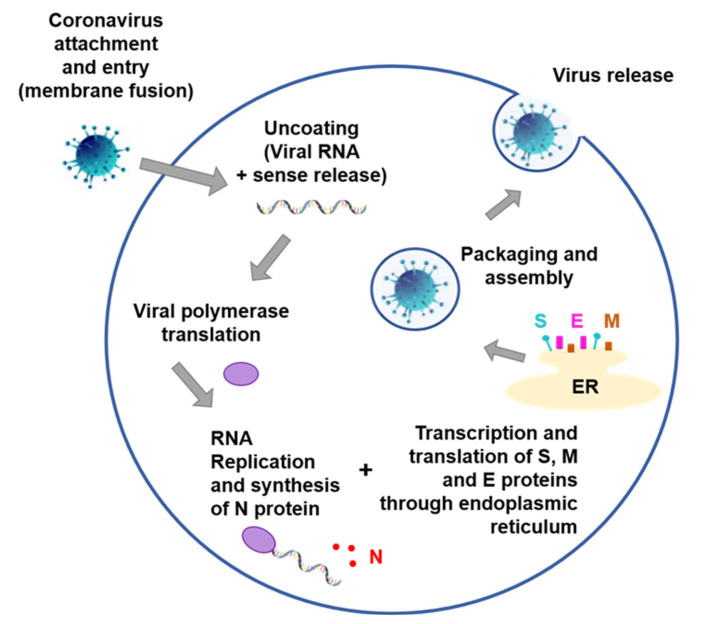
General human pathogenic coronavirus replication. Attachment and entry through S protein binding to specific host receptor. Positive sense viral RNA released and polymerase is translated. Viral RNA is replicated and nucleocapsid (N) structural protein is synthesized in the cytoplasm, and S protein, membrane (M) and envelope (E) are transcribed/translated in the endoplasmic reticulum (ER) and transported to the Golgi. Viral components are packed and assembly in a mature virion structure that is then released [4,24].

SARS-CoV-2 is an enveloped virus with glycoproteins in its surface, with positive-sense single-stranded RNA inside its protein capsid [21]. It is known that SARS-CoV-2 uses its spike protein (S) to attach to host cells through the angiotensin-converting enzyme 2 (ACE2), and it needs a protease, the TMPRSS2, to mediate the entry into the cell [22]. Once inside, the uncoated RNA is translated and replicated to form new virions (Figure 1). Comparing SARS-CoV-2 with SARS-CoV, both show typical *Betacoronavirus* gene structure, with remarkable protein homology, with 75% similarity of spike protein, including a mutation in the C-terminal RBD for enhanced binding to ACE2 [23].

Although the mutation rate of SARS-CoV-2 is lower than for Influenza virus, the emergence of variants of concern for SARS-CoV-2 has become frequent, representing a risk of resistance against the vaccines that are available. According to genomic analysis [25,26,27], variants that spread widely and showed evidence of increased transmissibility, increased severity, and reduced antibody neutralization of COVID-19 were classified as Variants of concern (VOCs) by the world health organization (WHO) and World Health Organization (WHO), US Center for Disease Control and Prevention (CDC) and COVID-19 Genomics UK Consortium (COG-UK). Of the analyzed variants, four were classified as VOCs, classified as B.1.1.7 (*Alpha*–Identified in UK), B.1.351 (*Beta*–Identified in South Africa), P.1 (*Gamma*–Identified in Brazil) and B.1.617.2 (*Delta*–identified in India) [27,28,29,30].

The increase in seroprevalence, transmissibility and evasion of the immune system caused by mutations in the virus created a fearful scenario mainly in the USA, Brazil, India, Russia and Mexico. Among these, underdeveloped countries like Brazil had very high rates and great difficulty in containing viral spread. In a global panorama, on 24 September 2021, about 230,706 cases were registered, of which 21,308 were diagnosed in Brazil, with an index of more than half a million deaths [31].

Adaptive mutations in the SARS-CoV-2 genome can alter its pathogenic potential and, at the same time, increase the difficulty of developing drugs and vaccines. Pharmacological means were gradually discovered and introduced, such as vaccines and monoclonal antibodies. Although it is difficult to find studies with nanomaterials focused on infections caused by Coronaviruses, this science is taking on greater visibility now due to the pandemic that the world is going through.

## 3. Metal Nanoparticles as Antivirals

The main strategies in the development of antivirals include the perception of viral structures and viral replication processes, making it possible to point out potential antiviral targets. Another possibility is the use of antivirals to strengthen the immune response to viral infection. The mechanism of one antiviral can vary as the target changes [4].

Among metallic nanoparticles, AgNPs are one of the best antimicrobials in efficiency against bacteria and viruses (Table 1) [32] because they are effective against several microorganisms even at a low concentration [33]. Lv and collaborators [34] tested different silver nanocomposites (20 nm AgNPs, 60 nm silver nanowires, 400 nm silver nanowires, and 10 nm silver colloids) with coated with Polyvinylpyrrolidone (PVP) against TGEV, porcine coronavirus, as a model of CoV. They demonstrated that all the nanomaterials, excepting silver colloids, showed an antiviral and inhibitory effect on TGEV entry and on apoptosis caused by the virus.

The antiviral effect of AgNPs is also showed by Chen and collaborators [35], inhibiting infection of feline coronavirus (FCoV) by blocking the entry to the host cells by physical biding. As other works demonstrated action of graphene oxide (GO) against coronaviruses (porcine epidemic diarrhea virus–PEDV) [36], Chen used nanocomposites of GO sheets along with silver against FCoV. They also address that due to the surface area/volume ratio and the possibility of tuning chemical and physical properties, no cytotoxicity is even presented at high concentrations. AgNP sized 11–12 nm from biological synthesis, using curcumin [32], also blocks the entry of RSV into Hep-2 cells, with nanoparticles possibly interacting with viral surface glycoproteins, and it is less toxic than AgNPs synthesized with citric acid in a chemical way.

Morris and collaborators [37] demonstrated the antiviral capacity of 8–12 nm AgNP, inoculated intranasally, against RSV during infection in vivo in BALB/c mice. Their suggestion of antiviral mechanism corroborates with the hypothesis of Yang and collaborators’ [32] report, through interaction of AgNP with RSV surface glycoproteins, inhibiting attachment of the virus to host cells. Silver nanoparticles with less than 5 nm in size were reported having antiviral effect against HIV, binding to the gp120 protein and affecting adsorption of the virus to CD4 host cell [38]. Smaller nanoparticles have a larger surface area/volume ratio; thus they are more prone to enter tissues and cells and to have better interaction with de virus; however, this penetration power can damage the host cells and cellular components, leading to a nanoparticle cytotoxicity increase [39].

Other metallic nanoparticles such as cooper nanoparticles also demonstrated antiviral activity. Fujimori and collaborators [40] showed antiviral activity of CuI nanoparticles against Influenza A virus (H1N1), inactivating the virus through generation of OH capable of degrading viral proteins, in a concentration-dependent manner. In their review, Sportelli and collaborators [41] affirm that coronaviruses are inactivated in copper surfaces due ROS created by copper ions release. Still, against H1N1 virus, zinc-oxide nanoparticles (ZnO-NPs) have antiviral activity after H1N1 infection to Madin-Darby canine kidney cells (MDCK-SIAT1), generating zinc ions and ROS that may potentially damage the host cell [42]. This toxicity can be decreased with surface coating with polymeric materials as polyethylene glycol as proposed to Ghaffari and collaborators [42].

Recently, a molecular docking of IO-NPs (Fe_2_O_3_ and Fe_3_O_4_) was performed to explore the interaction with the S protein receptor binding domain of SARS-CoV-2 (S1-RBD) [43]. As the interaction of Fe_3_O_4_ with S1-RBD involved the formation of four hydrogen bonds, it was considered stable and complex, and in addition, hydrophobic interactions of this IO-NP were detected with Leu455, Ser494 and Phe497 in the active site of S1-RBD, which could hinder virus adsorption to host cells [43]. Another approach with IO-NPs was validated against H1N1 influenza virus, where the 10 to 15 nm iron oxide nanoparticles (IO-NPs) show that may bind to the virus and inhibit its adsorption [44].

Gold is extensively used for diagnostic devices and vaccines for being chemically inert and relatively nontoxic [45]. Zacheo and collaborators [46] successfully tested gold nanoparticles (AuNPs) sized 4–5 nm with sulfonated group ligands varying in number, size, orientation and the sugar as head groups against Dengue virus (DENV). With a moderate toxicity and virucidal effect, a complex multi-sulfonated with glucose as head group was found to be the most effective against DENV-2, suggesting that this complex of AuNP interacts with DENV-2 envelope protein, inhibiting the virus permanently. AuNPs can also break disulfide bonds of the highly conserved protein Hemagglutinin (HA) of influenza viruses. Kim and collaborators [47] synthesized porous AuNPs without surfactant that decrease infectivity of various influenza virus strains (H1N1, H3N2 and H9N2). These strategies are promising and should be considered when studying antiviral approaches against SARS-CoV-2 because hemagglutinin esterase is a structural glycoprotein of some coronaviruses [48].

**Table 1:** Studies of nanometallic materials applied as antivirals reported in this review.

Nanomaterial	Virus Tested	Suggested Mechanism of Action	References
AgNPs and silver nanowires	TGEV	Inhibitory effect on TGEV infection and multiplication. Cell apoptosis reduction (activation of p38/mitochondria-caspase-3 signaling).	Lv et al., 2014 [34]
Nanocomposite Ag + Graphene oxide (GO-Ag)	FCoV	Inhibition of infection through interaction of GO with viral envelope and Ag binding to viral proteins	Chen et al., 2016 [35]
AgNP functionalized by Curcumin (cAgNP)	RSV	Reduce the binding ability of the virus on the cell surface.	Yang et al., 2016 [32]
AgNPs	RSV	Interaction with RSV surface glycoproteins, inhibiting attachment of the virus to host cells.	Morris et al., 2019 [37]
AgNPs	HIV	Binding to the gp120 protein and affecting adsorption of the virus to CD4 host cell	Singh et al., 2019 [38]
CuI-Nps	H1N1	Generation of ·OH, degrading viral proteins	Fujimori et al., 2011 [40]
ZnO-NPs	H1N1	Generation of zinc ions and ROS	Ghaffari et al., 2019 [42]
IO-NPs	SARS-CoV-2	Binding to S1-RDB to hinder virus adsorption (docking study)	Abo-zeid et al., 2020 [43]
IO-NPs	H1N1	Binding to the virus and inhibit virus adsorption to host cell	Kumar et al., 2019 [44]
Multi-sulfonated AuNPs	DENV	Interaction with envelope protein, inhibiting the virus permanently	Zacheo et al., 2020 [46]
AuNPs	H1N1, H3N2 and H9N2	Breakage of disulfide bonds of Hemagglutinin (HA) of influenza viruses	Kim et al., 2020 [47]

## 4. Nanoparticulated Delivery Systems against Viruses

Many medicines, including antiviral drugs marketed today, have issues that decrease their effectiveness, such as difficulties with solubility, permeability and absorption, affecting the drug bioavailability. Consequently, it is necessary to administer intravenous high doses to increase the frequency of medication administration or prolonged treatment duration, which may negatively affect the patient leading to major adverse effects. As a solution, nanoparticulated delivery systems can overcome these issues by improving the action of existing drugs, carrying bioactive compounds, immunogenic drugs or proteins that stimulate the host immune system [49].

Nanocarriers are well studied against HIV, improving the spreading of already anti-HIV used drugs in different tissues [50,51]. Because there have been no known drugs until now that effective interfere with SARS-CoV-2 replication, this type of strategy—drug nanocarrier—is not addressed in this work.

On the other hand, nanoparticulated systems can also carry molecules that somehow may interfere with viral replication. Aiming specific Dengue virus genes, Paul and collaborators [52] investigated the antiviral efficacy of biocompatible gold nanoparticles carrying small interfering RNA in a cationic complex AuNP-siRNA capable of inhibiting the replication of the dengue virus (DENV) in vitro. The complex interferes with the viral entry due its viral surface positive charging, binding to the host’s negatively charged cell membrane. This linkage releases siRNAs constructed to attach to the genes responsible for the expression of capsid or β-actin, and the decreased expressions were verified by qPCR, plaque forming assay and an immunostaining assay [52]. This strategy can be studied for usage against coronaviruses and other emergent microorganisms; however, in vivo testing is necessary, considering aspects as size regulation, configuration, and surfaces modification to achieve the best balance between host toxicity and antiviral effectiveness.

## 5. Nanovaccinology

Vaccination is the most effective medical intervention when it comes to severe viral infections, and to date, many vaccines have been developed against COVID-19 infection using various approaches (subunit vaccines, viral vector vaccines, and DNA vaccines). Nanoparticles are extensively explored, such as vaccine adjuvants due mainly to three essential characteristics: 1. can expose increased levels of antigen to the immune system; 2. capacity of controlled antigen-delivery, boosting antigen availability over time, guaranteeing an immune response; 3. competence to combine delivery with modulation or stimuli of the immune system. Most common nanoparticulated systems in vaccinology are lipid-based and polymeric nanomaterials [53,54].

A popular inorganic/metallic nanomaterial used for vaccination are gold nanoparticles (AuNPs) with advantageous physicochemical characteristics, low toxicity and simple obtention (synthesis) [55,56]. In 2011, Staroverov and collaborators evaluated the protective immune response stimulated by injection of TGEV-conjugated AuNPs in immunized mice and rabbits. TGEV-conjugated colloidal gold demonstrated higher concentrations of IFN-γ and neutralizing antibody in vaccinated animals. Besides that, the immunization with the complex TGEV + AuNPs increased propagation of T cells tenfold in comparison with the treatment without the antigen (TGEV), a promising result regarding the use of the virus associated with the gold nanoparticles for the development of vaccines [57].

Chen and collaborators [35] conjugated 100 nm gold nanoparticles with S (spike) glycoprotein of avian coronavirus and tested the vaccine in vivo demonstrating a strong IgG response and immunization of mouse. The S protein was also used in vaccination with gold nanoparticles in mouse in a study of Sekimukai and collaborators [58], displaying a strong IgG response but failing in decrease or avoiding allergic inflammatory response of SARS. Chen and collaborators [24] propose a vaccine that unites the immunomodulation of AuNPs, capped with polysaccharide that has antiviral properties, loaded with S or N proteins from SARS-CoV-2.

Very few nanometallic-based pharmaceuticals are marketed today, mainly due to toxicity and issues with biodegradation, limiting the usage of metal nanoparticles in infectious diseases treatment [59]. In order to mitigate the risks of nanotoxicity, some solutions can be adopted, such as surface modification through the use of agents such as poly(lactic-co-glycolic acid) (PLGA) or the biological synthesis of these NPs, which can facilitate the immune response generated by NP and increase the specificity for target tissues [60]. Besides that, AuNPs interest by its bio-inertness and low toxicity, which can be a potential candidate for adjuvant tool in the development of vaccine against the new coronavirus [55].

## 6. Metallic Nanoparticles in COVID-19 Diagnosis

COVID-19 early diagnosis is fundamental to contain development of the patient’s disease, also to avoid SARS-CoV-2 transmissibility and to manage the pandemic to its final course, especially at the time when there were not vaccines available. In order to develop low-cost approaches, seeking fast, accessible and trustworthy SARS-CoV-2 detection, research in novel devices and methods to detect the virus in different patient samples increased significantly in the past two years. In this context, nanotechnology is undoubtedly a weapon in the fight against COVID-19 [61].

COVID-19 diagnosis relies on viral gene sequences, patients’ antibodies or SARS-CoV-2 antigens detection from nasopharyngeal or oropharyngeal swab sample from patients (the gold standard of sampling) [62]. Combining diagnosis with the ability to tailor a metallic nanomaterial with a specific size and shape and high surface-to-volume ratio opens possibilities to design various biosensing methods. Gold nanostructures and their optical properties, for example, were already exploited in SARS-CoV-2 detection within a chip where these nanomaterials were functionalized with DNA receptors, and when hybridized with antigen nucleic acid, the combination of plasmonic photothermal and the surface plasmon resonance could be read by a laser beam, leading to an accurate detection [63].

Nanogold is also exploited in naked eye colorimetric assays, as well as lanthanide with its luminescence properties, detecting antigens from the virus or antibodies from the patient [63]. These naked-eye type detections are useful in point-of-care testing where the detection system is assembled in accessible and uncomplicated kits or paper-like membrane/lateral flow strip, enabling the home diagnosis [64].

Magnetic nanoparticles bring easy and effective possibilities in SARS-CoV-2 detection, through electrochemical, fluorescence or magnetic resonance properties [63]. Nucleic acid extraction, an important part of for COVID-19 diagnosis based on nucleic acid detection strategies, can be simplified through SARS-CoV-2 RNA separation with magnetic nanoparticles coated with carboxyl polymer, increasing the sensitivity of detection based on amplification methods [64].

Giovannini, Haick and Garoli (2021) [62] brought in their study a distinguished method to diagnosis COVID-19 from exhaled breath condensate (breath’s liquid phase), where the detection can take place in an electrochemical device build with modified gold nanoparticles where the patient is asked to cough or exhale with the month for 30 min aiming the device. Detection is possible due differences in electrical resistances between comparison of three samples: from sick and cured patients, and a control sample.

## 7. Metallic Nanoparticles in Personal Protective Equipment

Even after the start of immunization of the population with the different vaccines already available, the use of personal protective equipment is still mandatory in many countries, and fortunately, there are reports that demonstrate the effectiveness of incorporating metal nanoparticles in such equipment. Additionally, although the results against SARS-CoV-2 are promising results, it is a fact that nanometals present potential cytotoxicity that still needs improvements, and a possibility is through refinement of parameters such as size, shape, surface charge and coating material to reach ideal biocompatibility and biosafety matters [45].

The versatility of nanomaterials for use in protective equipment is wide and represents a simple way to contain viral spread and this technology has already been discussed in the use of silver nanoparticles [65], copper oxide [66], iodine [66,67], titanium oxide [66].

Ahmed and collaborators [68] developed a reusable, antimicrobial and antiviral face mask through the incorporation of a filtration system composed of a polylactic acid and cellulose acetate nanofibrous matrix containing copper oxide nanoparticles (CuONPs) and graphene oxide nanosheets and produced by the electrospinning technique.

Cento and collaborators [69] bring in their article applications of AgNPs for the prevention of respiratory diseases through usage of these nanos in a biogel applicated in nasal filters as PPE that can be utilized in hospital, domestic or outdoor environments. This biogel-AgNP was capable to inactivate bacteria (*Escherichia coli*) and virus (influenza virus A/PR/8/34), demonstrating a great potential to reduce the transmission of potential respiratory pathogens. As suggested in the title of Uskokovic’s [70] article “why have nanotechnologies been underutilized in the global uprising against the coronavirus pandemic?”, we must take better advantage from metallic nanoparticles in prevention, using them in personal protection equipment, fortifying our shields against the virus [41].

## 8. Conclusions and Futures Perspectives

Because viruses are obligate intracellular parasites and use the host-cell machinery for replication, it is hard to develop an antiviral drug with the capacity to target specific virus metabolic functions without injury the host. Considering the tunable physical and chemical characteristics, nanotechnology is a potential candidate in fighting the actual pandemic challenge imposed upon the medical field, reflecting consequences in all society sectors.

Although the results presented on these articles are extremely encouraging to replace currently used antivirals that are known to have major side effects, further experimental confirmations are necessary to manage nanomaterials as antivirals, especially in vivo. Therefore, as their properties can be easily adjusted by the synthesis process, more elaborate analyses of the behavior of nanocomposites in blood must be carried out in order to better understand the long-term toxicokinetics of nanoparticles in the human body.

Furthermore, strategies for the large-scale production of metallic nanoparticles must be considered. Its use as a prevention tool against future epidemics or pandemics can be widely explored because, in addition to the fact that they are incorporated in PPE, they can be incorporated in disinfectants to mitigate viral spread [71,72]. In addition to its application in this area, studies on the use of nanoparticles as tools aimed at improving drug delivery can help in the improvement of conventional therapies and vaccines.

Therefore, the versatility of nanoparticles is an important factor for their incorporation in several strategies to face not only the COVID-19 pandemic, but also to drive new therapeutic approaches and encourage the use of nanocomposites in the management of future pandemics.

## Data Availability

All data reported in this paper is from articles openly available on PubMed (nih.gov) (accessed on 10 October 2021).
